# Concordance between the Clinical Diagnosis of Influenza in Primary Care and Epidemiological Surveillance Systems (PREVIGrip Study)

**DOI:** 10.3390/ijerph19031263

**Published:** 2022-01-23

**Authors:** Carina Aguilar Martín, Mª Rosa Dalmau Llorca, Elisabet Castro Blanco, Noèlia Carrasco-Querol, Zojaina Hernández Rojas, Emma Forcadell Drago, Dolores Rodríguez Cumplido, Alessandra Queiroga Gonçalves, José Fernández-Sáez

**Affiliations:** 1 Primary Care Intervention Evaluation Research Group (GAVINA Research Group), IDIAPJGol Terres de l’Ebre, 43500 Catalonia, Spain; caguilar.ebre.ics@gencat.cat (C.A.M.); rdalmau.ebre.ics@gencat.cat (M.R.D.L.); zojahernandez@gmail.com (Z.H.R.); eforcadellg.ebre.ics@gencat.cat (E.F.D.); drficf@gmail.com (D.R.C.); aqueiroga@idiapjgol.info (A.Q.G.); jfernandez@idiapjgol.info (J.F.-S.); 2 Terres de l’Ebre Research Support Unit, Foundation University Institute for Primary Health Care Research Jordi Gol i Gurina (IDIAPJGol), 43500 Catalonia, Spain; 3 Unitat d’Avaluació, Direcció d’Atenció Primària Terres de l’Ebre, Institut Català de la Salut, 43500 Catalonia, Spain; 4 Equip d’Atenció Primària Terres de l’Ebre, Institut Català de la Salut, 43500 Catalonia, Spain; 5 Campus Terres de l’Ebre, Universitat Rovira i Virgili, 43500 Catalonia, Spain; 6 Unitat de Recerca, Gerència Territorial Terres de l’Ebre, Institut Català de la Salut, 43500 Catalonia, Spain; 7 Institut Català de la Salut, Hospital Universitari de Bellvitge, 08907 Catalonia, Spain; 8 Unitat Docent de Medicina de Familia i Comunitària, Tortosa-Terres de l’Ebre, Institut Català de la Salut, 43500 Catalonia, Spain

**Keywords:** influenza, public health surveillance, epidemics

## Abstract

Introduction: Health authorities use different systems of influenza surveillance. Sentinel networks, which are recommended by the World Health Organization, provide information on weekly influenza incidence in a monitored population, based on laboratory-confirmed cases. In Catalonia there is a public website, DiagnostiCat, that publishes the number of weekly clinical diagnoses at the end of each week of disease registration, while the sentinel network publishes its reports later. The objective of this study was to determine whether there is concordance between the number of cases of clinical diagnoses and the number of confirmed cases of influenza, in order to evaluate the predictive potential of a clinical diagnosis-based system. Methods: Population-based ecological time series study in Catalonia. The period runs from the 2010–2011 to the 2018–2019 season. The concordance between the clinical diagnostic cases and the confirmed cases was evaluated. The degree of agreement and the concordance were analysed using Bland–Altman graphs and intraclass correlation coefficients. Results: There was greater concordance between the clinical diagnoses and the sum of the cases confirmed outside and within the sentinel network than between the diagnoses and the confirmed sentinel cases. The degree of agreement was higher when influenza rates were low. Conclusions: There is concordance between the clinical diagnosis and the confirmed cases of influenza. Registered clinical diagnostic cases could provide a good alternative to traditional surveillance, based on case confirmation. Cases of clinical diagnosis of influenza may have the potential to predict the onset of annual influenza epidemics.

## 1. Introduction

Influenza is an infectious disease caused by RNA viruses of the genera Alphainfluenzavirus and Betainfluenzavirus (family: Orthomyxoviridae). It is responsible for high morbidity and high mortality in risk groups every year during the cold season [1]. The minimum surveillance required by the World Health Organization (WHO) consists of collecting weekly data based on individual cases or cases aggregated at the country level, at least during the period of the epidemic. These data can be of suspected and/or laboratory-confirmed cases [2]. Specifically, the WHO recommends surveillance through systems based on networks of sentinel physicians. A network of sentinel physicians is a group of health service physicians who collect respiratory samples from patients with symptoms suggestive of acute respiratory diseases (including influenza and its subtypes and lineages); the laboratory results that arise from this are reported in accordance with WHO criteria. A network of sentinel physicians supply updated information during the weeks of epidemic risk of the frequency, severity, and potential for an epidemic of the reported diseases. In addition, the WHO facilitates the exchange of information and biological material between countries through the Global Influenza Surveillance and Response System program [3]. It also promotes the dissemination of countries’ surveillance data to the public through the FluNet database, in this way making it possible to carry out global surveillance of influenza [4]. In the wake of the SARS-CoV-2 pandemic, the WHO advises that flu surveillance systems be maintained and expanded to include surveillance of the SARS-CoV-2 virus [5].

In Spain, the networks of sentinel physicians of the autonomous communities notify the Spanish Influenza Surveillance System about the number of confirmed cases from the samples collected. All cases confirmed outside the network of sentinel physicians (non-sentinel information) are also reported to the Spanish Influenza Surveillance System [6].

Worldwide, several countries have compared their syndromic surveillance data with confirmed cases, generally obtaining good correlation or concordance. A study in the USA found a good correlation between sales of antivirals and the frequency of confirmed cases, and between sales of antivirals and the proportion of general practitioner visits due to influenza-like illness [7]. In South Korea, correlations were found between positive cases identified with rapid antigen tests and the proportion of general practitioner visits due to influenza-like illness, and between cases confirmed by rapid antigen tests and cases confirmed by RT-PCR [8]. Correlations were also noted between the cases reported to the mandatory disease notification system and the proportion of positives, and between the proportion of general practitioner appointments due to influenza-like illness and the number of influenza-like illness cases in China [9]. In Spain, based on regional data, concordance was observed between the cases recorded in patients’ clinical histories and the cases confirmed by the network of sentinel physicians of the Balearic Islands [10]. Another study found a correlation between the cases confirmed by the network of sentinel physicians and severe hospitalized cases of laboratory-confirmed influenza in Catalonia [11]. In Europe, countries such as Poland have a double system of survey reporting the incidence of both clinically and laboratory-confirmed influenza and influenza-like illnesses and also acute respiratory tract infections, according to the criteria set for the surveillance of influenza in the European Union [12]. In Spain, with data from other regions, concordance has been observed between the cases recorded in the clinical history and the cases confirmed by the sentinel physician network (Balearic Islands) [10]. Additionally, in another study, the correlation between the cases confirmed by the sentinel physician network and the serious hospitalized cases of confirmed influenza (Catalonia) [11] was evidenced.

In Catalonia (Spain), the network of sentinel physicians publishes weekly results at least one week in arrears from the day of disease registration. This, and the annual fluctuation at the start of the influenza epidemic, are obstacles to the management of health services and to effective clinical approaches. However, clinical diagnostic data are published within a week of registration. If a high concordance between the clinical and confirmed diagnostic data is found, they could be used to monitor the evolution of the epidemic in something closer to real time, which would enable its management to be optimized. Therefore, the objective of this study is to assess whether there is concordance between the number of clinically diagnosed cases and the number of confirmed cases of influenza.

## 2. Materials and Methods

### 2.1. Design and Study Population

We carried out a population-based ecological time series study, using the number of clinically diagnosed cases of influenza, cases of influenza confirmed by the network of sentinel physicians, and total confirmed cases of influenza in Catalonia. The study period ran from the 2010–2011 season to the 2018–2019 season. Within each year, the period considered to be the influenza season, from week 40 of a given year to week 20 of the next, was studied.

### 2.2. Data Collection

The data collected were secondary and public. The reference population was that of the autonomous community of Catalonia (Spain).

The weekly number of clinically diagnosed influenza cases was obtained from the DiagnostiCat website for each season from 2010 to 2019 [13].

DiagnostiCat collects the clinical diagnoses of all primary care physicians who use the eCAP medical records program. These physicians include all those from the Institut Català de Salut and some others who work in Sistema sanitari integral d’utilització pública de Catalunya (SISCAT) and other Catalan health service institutions who use eCAP [14].

The data on confirmed sentinel cases of influenza were obtained from the network of sentinel physicians of Catalonia, which publishes them in the Pla d’Informació de les Infeccions Respiratòries Agudes a Catalunya (PIDIRAC) [15]. Each sentinel physician collects a sample, consisting of throat and nasal swabs, of the first two cases they see every week that are consistent with influenza syndrome and sends them to the laboratory to confirm whether there is indeed an influenza virus infection [16]

Following the European definition of an influenza case [17], the criteria for clinical suspicion of influenza used by network of sentinel physicians are the following [16]: sudden onset of symptoms; at least one of four general symptoms—fever or low-grade fever, general malaise, headache, myalgia; at least one of three respiratory symptoms—cough, odynophagia, dyspnoea; absence of any other suspected diagnosis.

The total confirmed cases of influenza include the cases confirmed by the sentinel physicians and those confirmed in all the healthcare facilities in Catalonia (primary care, hospitals, and other institutions), in other words, those not in the network of sentinel physicians (non-sentinel information) [6]. The total confirmed cases of influenza data were extracted from the Spanish Influenza Surveillance System website [18] using the WebPlotDigitizer tool [19].

Therefore, DiagnostiCat includes cases confirmed by the network of sentinel physicians. Total confirmed cases of influenza include cases confirmed by the network of sentinel physicians. Total confirmed cases of influenza are not all included in DiagnostiCat because most of them are influenza cases confirmed in hospital.

### 2.3. Statistical Analysis

The numbers of cases of clinical diagnosis, confirmed sentinel cases, and total confirmed cases of Influenza were collected, and the following rates were calculated: clinical diagnosis rate—number of weekly clinical diagnostic cases with respect to the population attended by Institut Català de Salut physicians, per 100,000 inhabitants; confirmed sentinel case rate—the number of cases confirmed by the network of sentinel physicians with respect to the population assigned to them, per 100,000 inhabitants; total confirmed influenza case rate—number of confirmed cases in all healthcare facilities in Catalonia with respect to the total population of Catalonia, per million inhabitants.

To evaluate the concordance between the clinical diagnosis and confirmed cases, the intraclass correlation coefficient of absolute agreement (ICC_A_) and consistency (ICC_C_) for single measures, and their corresponding 95% confidence intervals (95% CIs), were calculated. ICCs were calculated for the rates of all three variables together and in pairwise combinations. The pairwise comparisons made were the following: clinical diagnosis with confirmed sentinel cases, and clinical diagnosis with total confirmed cases of influenza.

The degree of agreement between the rates of clinical diagnosis and confirmed cases was evaluated by the Bland–Altman graphical method [20,21] for both comparisons. This method plots the means of the two rates against the difference between them. When a proportional relationship was found in the representation of the original rates, a logarithmic transformation was carried out [21], and the Bland–Altman graphs replotted.

In Bland–Altman graphs, the mean of the differences between the two methods indicates the systematic error that exists between the clinical diagnostic rate and the rate of confirmed cases. It indicates the extent to which the clinical diagnosis produces underestimates or overestimates with respect to the rate of confirmed cases. The more tightly clustered the points are around the mean, the greater the precision of the estimate. The limits of agreement allow the difference between the two systems to be quantified. The estimators of the graph with the logarithmically transformed data are interpreted as a function of the percentage of variation.

Pairwise Pearson and Spearman correlation coefficients were also calculated (clinical diagnosis with confirmed sentinel cases, and clinical diagnosis with total confirmed cases of influenza).

Statistical analyses were performed with R (version 3.5.2) [22].

## 3. Results

The results from the nine study seasons, from 2010–2011 to 2018–2019 (i.e., prior to the COVID-19 pandemic), are shown.

The overall concordance between the rates of clinical diagnoses, confirmed sentinel cases, and total confirmed influenza cases obtained for all the seasons is illustrated by the ICC_A_ of 0.470 (95% CI, 0.332–0.588) and the ICC_C_ of 0.539 (95% CI, 0.475–0.601). The degree of agreement and concordance was similar in all seasons and no trend in the ICC values was observed. The maximum ICCs noted were between the rates of clinical diagnosis and total confirmed influenza case in the 2018–2019 season (ICC_A_ = 0.796 and ICC_C_ = 0.836). The minimum values were obtained in the 2012–2013 season for the same comparison of rates (ICC_A_ = 0.159 and ICC_C_ = 0.206) (Table 1).

**Figure 1:**
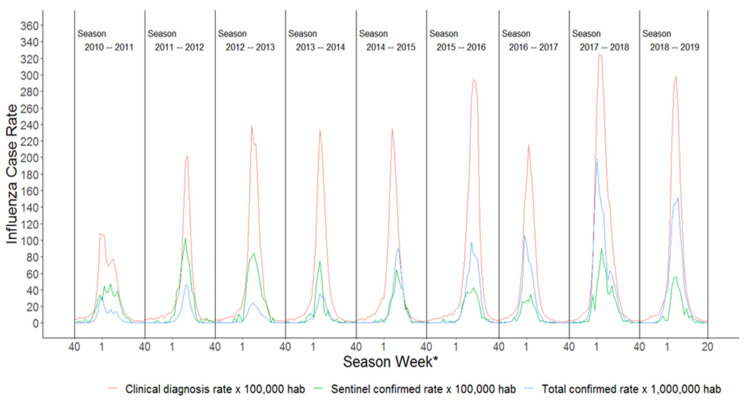
Clinical diagnostic rates, sentinel-confirmed rates, and total case rates. * Each season is represented from week 40 of the 1st year to week 20 of the 2nd year.

The ICC values before 2014–2015 were higher for the rates of clinical diagnosis with confirmed sentinel case rates, and after, the concordance was higher for the rates of clinical diagnosis with total confirmed influenza case rates. In the 2014–2015 season itself, the concordance between clinical diagnosis rates and total confirmed influenza case rates, clinical diagnosis rates, and confirmed sentinel case rates were similar (Table 1 and Figure 1).

The data are presented as graphical representations of the weekly rates, in which it can be seen that the epidemic curves of confirmed sentinel cases, total confirmed influenza cases, and clinical diagnosis are similarly shaped in terms of the weeks of the rise, peak, and fall, showing parallelism between them (Figure 1). The confirmed sentinel rate ranged from 0 to 108.8 per 100,000 inhabitants. The total confirmed influenza rates increased over the seasons. The maximum rate was recorded in the 2017–2018 season, with 198.9 confirmed cases per 1,000,000 inhabitants. The rates for the 2010–2011 season were the lowest for confirmed sentinel cases, total confirmed influenza cases, and clinical diagnosis. Meanwhile, the 2015–2016, 2017–2018, and 2018–2019 seasons presented higher rates of clinical diagnosis, that of 2017–2018 having the highest incidence of those studied (Figure 1). The curve corresponding to the total confirmed influenza case rates shows that they started out lower than the confirmed sentinel case rates. As the seasons progressed, the total confirmed influenza case rate began to rise, while the confirmed sentinel case rate remained roughly the same. In the 2014–2015 season, the total confirmed influenza case rate exceeded the confirmed sentinel case rate, and remained higher until the end of the study period (Figure 1).

The values observed in the ICC (Table 1) coincide with the trends in the rates illustrated in Figure 1. In the 2010–2011 and 2013–2014 seasons, the confirmed sentinel case rate was higher and more akin to that of clinical diagnosis, which therefore gives rise to greater concordance. Between seasons 2015–2016 and 2018–2019 there was greater concordance between the rates of clinical diagnoses and the total confirmed influenza case rates (Figure 1).

**Figure 2:**
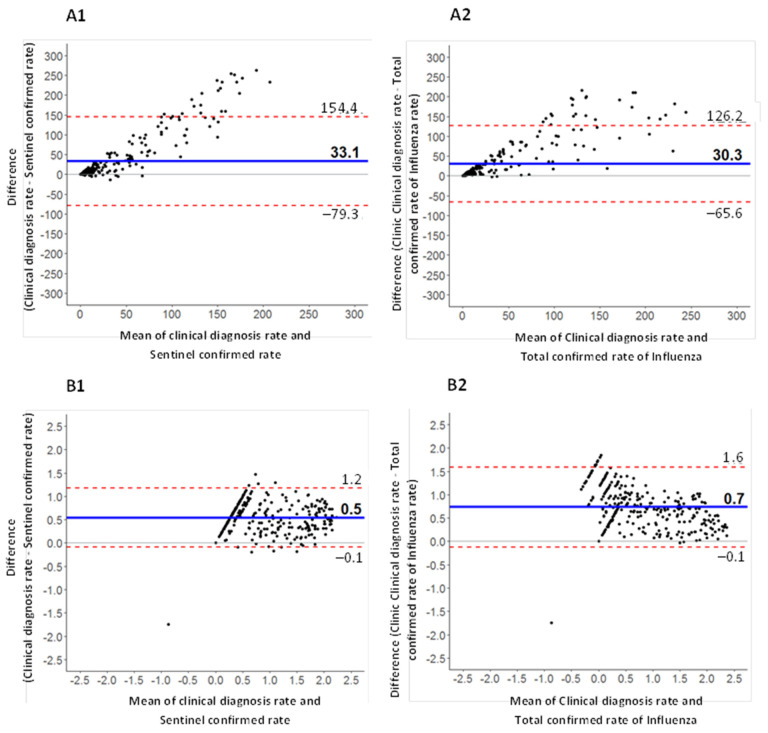
Bland-Altman graphs using data from all seasons studied. Figures (**B1**,**B2**) with logarithmic transformation. Figures (**A1**,**A2**) represent data before transformation. Figures (**B1**,**B2**) after applying logarithmic transformation.

The Bland-Altman graphs show that there was maximum agreement between the three surveillance systems at the beginning of the annual epidemic. Throughout the epidemic, the clinical diagnoses overestimated the rate of influenza with respect to confirmed sentinel cases rates and total confirmed influenza cases rates. Both graphs (Figure 2(A1,A2)) show an upward trend, confirming that more clinical diagnoses were made as the epidemic evolved. Moreover, the limits of agreement (upper and lower dotted lines) were closer in the comparison between the rates of clinical diagnosis and total confirmed influenza cases rates (Figure 2(A2)) than in the case of the clinical diagnosis and confirmed sentinel cases rates comparison (Figure 2(A1)). This indicates that the rate of clinical diagnosis was in closer agreement with the total confirmed influenza case rate than with the confirmed sentinel case rate.

After log transformation to correct the systematic error associated with the different magnitudes of the three surveillance systems (the clinical diagnosis always had higher absolute values and rates) the concordance was confirmed (Figure 2(B1,B2)).

The Pearson correlation between the rates of clinical diagnoses and confirmed sentinel cases rates (*r* = 0.822) was lower than that between the clinical diagnoses rates and total confirmed influenza cases rates (*r* = 0.850) over the entire study period. The Spearman correlations were higher than the Pearson correlations, between both the clinical diagnoses rates and confirmed sentinel case rates (*ρ* = 0.864), and the clinical diagnoses rates and total confirmed influenza case rates (*ρ* = 0.891) (Table 2).

## 4. Discussion

The results of this study show that there was a high degree of concordance between the rates of clinical diagnosis and the confirmed cases of influenza rates.

From the 2015–2016 season to the 2018–2019 season, the concordance of the clinical diagnosis rates was greater with the total confirmed influenza case rates, while before the 2014–2015 season, the concordance was greater with the confirmed sentinel case rates. The biggest difference between the surveillance systems was seen when there were high rates of influenza. Conversely, the systems coincided when the rates of influenza were low. Therefore, the rates of clinical diagnosis (DiagnostiCat) could be used to predict the onset of the epidemic, since they were consistent with the rates of confirmed cases, especially at the beginning when the rates were low.

In general, the values of the ICC_C_ were higher than those of the ICC_A_. The ICC_A_ reached its maximum value of one when the measurements were equal and penalized any type of difference between the two measurements. On the other hand, the ICC_C_ was sensitive to the systematic differences of the different measurement systems. When there were few cases of influenza, the clinical diagnosis agreed with the confirmed influenza cases, since the rates were similar. However, as the epidemic progressed, the rate of clinical diagnoses increased more rapidly than the confirmed case rate. As the difference between the two systems was not constant, the value of ICC_C_ decreased.

The correlation study showed values that were higher than those of the concordance, as was expected.

A significant proportion of the studies published so far that compare systems of surveillance or monitoring of influenza activity have used the Pearson correlation coefficient [7,8,23,24] or the Spearman correlation coefficient [9,25,26]. A minority have adopted a method similar to that of the present study (ICC and/or Bland–Altman graphs) [10,25], since this is appropriate for comparing two measures and because Bland–Altman graphs are easy to interpret.

The correlation is not suitable in this case because DiagnostiCat partially includes the total confirmed cases of influenza and confirmed sentinel cases, which means that the correlation will be always high. Furthermore, it is worth remembering that the ICC is the method of choice for evaluating concordance with numerical variables.

A good level of agreement implies that the surveillance systems are interchangeable. When concordance is high, one measurement system can therefore be used instead of another. In this case, clinical diagnoses could be used instead of confirmed cases, since the clinical data are available sooner. After the COVID19 pandemic, the results from the confirmed cases might be a better choice as these two viral diseases share similar symptoms. Currently, in the primary care setting, rapid diagnostic tests are being implemented allowing differentiation SARS-CoV-2, Influenza A and B, respiratory syncytial virus, and adenovirus.

All surveillance systems have their own particular limitations. The number of samples sent by the network of sentinel physicians is partly determined by the number of collaborating physicians and vacation periods. Sentinel physicians’ vacations, and the fact that people are less likely to visit their GP during vacations leads to lower influenza rates than expected [27,28]. The total confirmed cases of influenza, when collecting all the cases confirmed by the network of sentinel physicians and from health services outside the network, are not limited with respect to the number of samples or confirmations. The progressive increase in total confirmed cases of influenza, which could be due to the reduction in the cost of laboratory tests [29,30], is striking. This increase could be responsible for the closer concordance between the clinical diagnosis and total confirmed cases of influenza.

DiagnostiCat was launched in 2010 in response to the need for information prompted by the 2009–2010 season influenza pandemic. It has recently incorporated new functions, for example, SeGrip. This webpage is updated daily to include each day’s new cases registered in the eCAP. It should be noted that four network of sentinel physicians health centres were not registered with eCAP until the 2018–2019 season [16].

The results emerging from the present study are comparable to those of studies that used similar methods. These studies revealed differences between the data from the network of sentinel physicians and other surveillance systems. The proportions of visits to primary care and 24-h services due to influenza-like illness in Australia were compared. There was good agreement between the two and, as in the present study, the differences between the rates of influenza noted by the two systems were greater because there were more cases of influenza (as the epidemic progressed) [31]. In the Balearic Islands (Spain), a high degree of concordance was found between the weekly rates of diagnoses recorded in the clinical history and the weekly rates of network of sentinel physician-confirmed cases [10]. The degree of concordance of our results was slightly lower than in the previous study, possibly due to the greater coverage by the network of sentinel physicians in the region where the study was conducted [6,10].

One of the limitations of the study is that, as a study using secondary data, it is not possible to be certain that the clinical diagnoses registered in DiagnostiCat were indeed confirmed. It should be remembered that the symptoms of influenza are the same as those of other respiratory diseases, for example, COVID-19 and common colds caused by other respiratory viruses. On the other hand, a small proportion of the doctors who are registered with the eCAP are not members of the Institut Català de la Salut. They are included in DiagnostiCat but not in the denominator of the clinical diagnosis rate, which means that the rates of clinical diagnosis could be overestimated. Another difficulty is that the clinical criteria for sample collection required for confirmation are not known in the case of the non-sentinel information included in the total confirmed cases of influenza. This is a concern as confirmed non-sentinel cases are increasingly being reported.

This study has considered data until the 2018–2019 season, as the COVID-19 pandemic subsequently hit. COVID-19 pandemic hindered the accurate discrimination of the two diseases, and then producing excess influenza cases that might in fact be COVID-19 [32]. Future studies should consider COVID-19, since it shares many symptoms with influenza; indeed, almost all the symptoms present in the definition of cases of influenza [33] are present in the description of suspected cases of COVID-19 [34].

The clinical diagnosis, and the greater ability to act in advance that it offers, could be key to predicting the onset of influenza in the future.

## 5. Conclusions

There is concordance between the clinical diagnosis and confirmed cases of influenza. Clinical diagnostic cases registered in DiagnostiCat could be a good alternative to traditional surveillance based on case confirmation. Data from DiagnostiCat could be used to predict the onset of the annual influenza epidemic as it is consistent with confirmed rates, especially when influenza rates are low. The main advantage of this system is the early publication of the number of cases, which allows more pre-emptive action to be taken to manage the annual influenza epidemic.

## Figures and Tables

**Table 1:** Intraclass correlation coefficient of absolute agreement (ICCa) and consistency (ICCc) with their confidence intervals 95% among the clinical diagnostic, sentinel-confirmed, and total confirmed cases.

	ICCa			ICCc		
Season	Clinical Diagnosis Rate vs. Sentinel-Confirmed Rate vs. Total Confirmed Rate	Clinical Diagnosis Rate vs. Sentinel-Confirmed Rate	Clinical Diagnosis Rate vs. Total Confirmed Rate	Clinical Diagnosis vs. Sentinel-Confirmed vs. Total Confirmed	Clinical Diagnosis Rate vs. Sentinel-Confirmed Rate	Clinical Diagnosis Rate vs. Total Confirmed Rate
2010–2011	0.460 (0.17–0.685) ***	0.592 (0.171–0.804) **	0.313 (−0.054–0.604)	0.587 (0.395–0.750) ***	0.687 (0.454–0.832) ***	0.448 (0.129–0.683) **
2011–2012	0.570 (0.334–0.749) ***	0.753 (0.505–0.878) ***	0.362 (0.026–0.626) *	0.639 (0.458–0.785) ***	0.789 (0.615–0.890) ***	0.438 (0.116–0.676) **
2012–2013	0.378 (0.150–0.596) ***	0.599 (0.262–0.793) ***	0.159 (−0.110–0.436)	0.452 (0.241–0.651) ***	0.661 (0.415–0.817) ***	0.206 (−0.142–0.509)
2013–2014	0.343 (0.122–0.565) ***	0.412 (0.062–0.666) *	0.243 (−0.056–0.519)	0.416 (0.202–0.623) ***	0.491 (0.182–0.711) **	0.305 (−0.037–0.584) *
2014–2015	0.410 (0.187–0.621) ***	0.323 (0.005–0.590) *	0.407 (0.079–0.656) **	0.472 (0.262–0.666) ***	0.383 (0.051–0.639) *	0.467 (0.152–0.696) **
2015–2016	0.378 (0.154–0.593) ***	0.226 (−0.064–0.500)	0.495 (0.137–0.726) **	0.450 (0.241–0.646) ***	0.281 (−0.058–0.562)	0.572 (0.295–0.761) ***
2016–2017	0.464 (0.227–0.670) ***	0.245 (−0.055–0.521)	0.661 (0.314–0.835) ***	0.539 (0.338–0.716) ***	0.306 (−0.036–0.585) *	0.726 (0.514–0.855) ***
2017–2018	0.553 (0.302–0.741) ***	0.348 (0.005–0.617) *	0.746 (0.425–0.883) ***	0.634 (0.452–0.782) ***	0.435 (0.112–0.674) **	0.802 (0.636–0.897) ***
2018–2019	0.552 (0.311–0.737) ***	0.297 (−0.019–0.569) *	0.796 (0.543–0.905) ***	0.624 (0.44–0.775) ***	0.365 (0.030–0.626) *	0.836 (0.693–0.915) ***
All	0.470 (0.332–0.588) ***	0.366 (0.160–0.522) ***	0.549 (−0.284–0.704) ***	0.539 (0.475–0.601) ***	0.434 (0.337–0.522) ***	0.626 (0.522–0.691) ***

* *p* < 0.05. ** *p* < 0.01. *** *p* < 0.001.

**Table 2:** Pearson and Spearman correlation coefficients between rates of clinical diagnosis, sentinel-confirmed, and total confirmed cases.

	Pearson Correlation Coefficient		Spearman’s Rank Coefficient	
Season	Clinical Diagnosis Rate vs. Sentinel-Confirmed Rate	Clinical Diagnosis Rate vs. Total Confirmed Rate	Clinical Diagnosis Rate vs. Sentinel-Confirmed Rate	Clinical Diagnosis Rate vs. Total Confirmed Rate
2010–2011	0.906 *	0.962 *	0.948 *	0.950 *
2011–2012	0.934 *	0.995 *	0.705 *	0.862 *
2012–2013	0.954 *	0.979 *	0.900 *	0.945 *
2013–2014	0.915 *	0.979 *	0.843 *	0.892 *
2014–2015	0.740 *	0.692 *	0.782 *	0.811 *
2015–2016	0.919 *	0.984 *	0.910 *	0.884 *
2016–2017	0.947 *	0.937 *	0.964 *	0.967 *
2017–2018	0.930 *	0.943 *	0.937 *	0.966 *
2018–2019	0.983 *	0.988 *	0.910 *	0.883 *
All	0.822 *	0.850 *	0.864 *	0.891 *

* *p* < 0.001.

## Data Availability

The data are secondary and public, DiagnostiCat website (https://www.ics.gencat.cat/sisap/diagnosticat/principal, accessed on 12 December 2021) collects the clinical diagnoses of all primary care physicians who use the eCAP [13]. The data on confirmed sentinel cases of influenza were obtained from the network of sentinel physicians of Catalonia, which publishes them in the *Pla d’Informació de les Infeccions Respiratòries Agudes a Catalunya* (PIDIRAC) [15]. The total confirmed cases of influenza data were extracted from the Spanish Influenza Surveillance System website (http://vgripe.isciii.es/PresentarGraficos.do, accessed on 12 December 2021) [18].
